# ASO Author Reflections: Laparoscopic versus Robotic Adrenalectomy in the Current Era

**DOI:** 10.1245/s10434-025-18699-3

**Published:** 2025-11-19

**Authors:** Eren Berber

**Affiliations:** Department of Endocrine and General Surgery, Cleveland Clinic, Cleveland, OH USA

## Past

The description of the laparoscopic technique by Gagner in 1992^1^ revolutionized the surgical approach to the adrenal gland, with data showing lower postoperative comorbidity and shorter length of stay with laparoscopic versus open adrenalectomy.^2^ Subsequently, the debates about adrenal surgery have been  mainly around the choice of laparoscopic lateral transabdominal versus posterior retroperitoneal approach  in a given patient, until robotic systems were introduced to general surgery about a decade later. Nevertheless, the early robotic surgical systems did not provide very significant advantages over laparoscopy to the surgeons, except for wristed instrumentation and three-dimensional (3D) view, due to the cumbersome docking mechanism, lack of a fourth arm, lack of fluorescence imaging, and excessive cost. In fact, the results of the first randomized study (*n* = 20) comparing laparoscopic versus robot-assisted adrenalectomy in 2004^3^ favored the laparoscopic approach, with a shorter operative time, less cost, and lower morbidity.

## Present

Nevertheless, the subsequent generation of robotic systems widened the gap with laparoscopy owing to the addition of a fourth arm, better 3D optics, indocyanine green fluorescence, articulating vessel sealers, and double console setup. With incorporation into endocrine surgical training programs, in addition to the increasing surgical experience, the adoption of robotic techniques in adrenal surgery accelerated, as evidenced by the 2016 National Inpatient Sample reporting that one-third of adrenalectomies in the USA were done robotically.^4^ As expected, the second randomized study comparing laparoscopic versus robotic adrenalectomy in 2020 reported better perioperative outcomes with the robotic approach for pheochromocytoma, albeit still with a higher cost.^5^ The current study compared laparoscopic versus robotic adrenalectomy (*n* = 27 each) in a more comprehensive fashion to include all diagnoses, not just pheochromocytoma, and also to account for the additional time required for the robotic setup (docking time) in the analysis.^6^ The results showed that perioperative outcomes, including cost, were similar between the two groups, with better ergonomics and lesser conversions from the original minimally invasive surgical plan in the robotic vs laparoscopic group. Although the study was not powered that way, the results were interesting in that, all of the robotic cases were completed in a minimally invasive fashion, but in the laparoscopic group, one patient each was converted to open, hand-assist, and partial adrenalectomy, and in another patient, the procedure was aborted , with the reason in all of these cases being the inability to continue the dissection safely and efficiently owing to challenges created by complex patient anatomy, tumor characteristics and difficult tissue planes that could not be overcome with rigid laparoscopic instruments and two-dimensional view.

## Future

What are the implications of the current randomized study?^6^ First of all, it suggests that in cases without significant challenges, such as large (> 5 cm) tumors, abundant perirenal fat (> 20 mm at the renal hilum), or periadrenal inflammation, both laparoscopic and robotic approaches could result in similarly excellent perioperative outcomes. However, in the presence of these challenging features, the robotic approachcould, most likely, provide a better chance to complete the procedure without any conversions or deviations. Nevertheless, in all scenarios, our results also showed that a less physical effort by the surgical team was necessary in robotic versus laparoscopic adrenalectomy procedures. On a busy surgical day, this benefit may have a significant positive effect on the well-being and performance of the surgical team, which has not been addressed much in the literature before. Nevertheless, with the heavy competition for robotic time by different specialties at most hospitals, it would be unrealistic to expect a robotic system to be available for all of the adrenalectomies in a program. Therefore, our suggestion for adrenal surgeons is to get familiar with both laparoscopic and robotic adrenalectomy techniques  and have the robot available for more complex cases to prevent conversions. We have started the second phase of the randomized study to provide more data on challenging adrenalectomies and hope to share it with the surgical community in the near future.
